# Poor return on investment: investigating the barriers that cause low credentialing yields in a resource-limited clinical ultrasound training programme

**DOI:** 10.1186/s12245-018-0168-9

**Published:** 2018-02-21

**Authors:** Hein Lamprecht, Gustav Lemke, Daniel van Hoving, Thinus Kruger, Lee Wallis

**Affiliations:** 1 Division of Emergency Medicine, Faculty of Medicine and Health Sciences, Stellenbosch University, PO Box 241, Cape Town, 8000 South Africa; 2 Department of Obstetrics and Gynaecology, Faculty of Medicine and Health Sciences, Stellenbosch University, Cape Town, South Africa

**Keywords:** Ultrasound, Training, Education assessment

## Abstract

**Background:**

Clinical ultrasound is commonly used in medical practices worldwide due to the multiple benefits the modality offers clinicians. Rigorous credentialing standards are necessary to safeguard patients against operator errors. The purpose of the study was to establish and analyse the barriers that specifically lead to poor credentialing success within a resource-limited clinical ultrasound training programme.

**Methods:**

An electronic cross-sectional survey was e-mailed to all trainees who attended the introductory clinical ultrasound courses held in Cape Town since its inception in 2009 to 2013. All trainees were followed until they completed their training programme in 2015.

**Results:**

Only one fifth of trainees (*n* = 43, 19.7%), who entered the Cape Town training programme, credentialed successfully. Ninety (*n* = 90, 41.3%) trainees responded to the survey. Eighty-six (*n* = 86) surveys were included for analysis. Time constraints were the highest ranked barrier amongst all trainees. Access barriers (to trainers and ultrasound machines) were the second highest ranked amongst the non-credentialed group. A combination between access and logistical barriers (e.g. difficulty in finding patients with pathology to scan) were the second highest ranked in the credentialed group.

**Conclusions:**

Access barriers conspire to burden the Cape Town clinical ultrasound training programme. Novel solutions are necessary to overcome these access barriers to improve future credentialing success.

**Electronic supplementary material:**

The online version of this article (10.1186/s12245-018-0168-9) contains supplementary material, which is available to authorized users.

## Background

The benefits of clinicians using ultrasound (clinical ultrasound, CUS) at the point of patient care have been well proven over the past 20 years [1–3]. It allows clinicians to produce additional diagnostic information at the patient’s bedside that is not assessable by physical examination alone. The benefits are enhanced in low- and middle-income countries (LMIC) where limited resources significantly restrict special investigation access [4]. However, if used poorly, it has the potential to contribute to misdiagnosis, needless downstream testing or treatment and possible patient harm. Rigorous training is needed to assure competency amongst CUS providers, to reduce operator errors that may lead to patient adverse events. Internationally, many clinical ultrasound training programmes exist, with slight variations in curricula content and delivery methods [5, 6]. In 2014, the International Federation for Emergency Medicine (IFEM) Ultrasound Special Interest Group published guidelines on how such a curriculum should be structured [7]. Nearly all training programmes follow the IFEM recommendations of starting with an introductory course, followed by completing a hands-on proctored scan list on real patients and finally a competency assessment to complete the credentialing process [7]. Certification should be provided to all successful candidates.

South Africa, a middle-income country, has a similar CUS training programme accredited by the Emergency Medicine Society of South Africa (EMSSA) and College of Emergency Medicine of South Africa (CEMSA) [8]. Doctors from any specialty and level are allowed to enter the training programme by attending an introductory course. Thereafter they are expected to gain experience by logging 65 scans on real patients, including patients with positive pathological findings (example: abdominal aorta aneurysm). Finally, trainees must pass a practical exit examination that consists of scanning live models and patients with real pathological findings. Trainees who eventually complete their credentialing are supplied with provider certificates and registered on the EMSSA web page to assure transparency of their competency status.

Training the curriculum on the traditional apprenticeship model (where certified trainers supervise and provide real-time feedback to trainees when scanning patients during the gaining experience phase) is an expensive use of already scarce resources. Not surprisingly, recent studies identified many barriers that negatively impact on trainees’ credentialing success in both LMIC and high-income country (HIC) settings [7, 9–13]. The process is severely time-consuming for both trainees and trainers. Trainee doctors must add the scanning requirements to their busy clinical schedules; the same challenge applies to the trainers who are responsible for providing feedback on the scans. However, none of the studies analysed the type of barriers that prevent credentialing in relationship to the training setting’s resources and most importantly their impact on the eventual credentialing success.

There is a need for data that focuses on the barriers in context to the training setting and credentialing outcomes. We undertook a study to establish and analyse the barriers that specifically lead to poor credentialing outcome in a resource-limited CUS training programme. The study results will provide valuable data to conceptualise future problem-solving research questions.

## Methods

### Study design

We undertook a cross-sectional study to conduct an electronic survey of trainees who entered the Cape Town CUS training programme. The study was performed from October 2013 to November 2015.

### Study setting

The trainee’s attendance at the introductory course marks their entry into the training programme. The required 65 scans were completed under certified trainers’ supervision in central academic, regional and district hospitals located in Cape Town, South Africa. Upon completion, trainees should pass the CUS exit examination within a 2-year period to successfully credential. Those who fail to complete their training within the required period are obliged to re-enter the programme by repeating the introductory course [8].

Cape Town is one of three national training centres responsible for providing the prescribed training curriculum and kept a database of all CUS trainees since inception of the programme on 1 June 2009.

### Study population

All trainees who attended the Cape Town training centre’s introductory course between 1 June 2009 and 30 June 2013 were eligible to partake in the study. None of the other two training centres kept databases of their course attendees that could have enhanced the study’s sample size.

The survey was conducted in October 2013 and the trainees were followed until 2015 to determine whether they successfully credentialed as CUS providers (credentialed group) or not (non-credentialed group) within the required 2-year period limit.

### Data collection and management

Trainees were invited by e-mail to complete an online questionnaire (Additional file 1). Their participation implied consent. Non-responders were reminded at 1-week intervals until they responded or the submission deadline expired after 1 month. No personal or identifying information was collected to protect participant confidentiality. The online survey platform de-identified all responses before converting the data into an Excel® electronic spreadsheet. The electronic spreadsheet was password protected to ensure the integrity of the data. The Health Research Ethics Committees at Stellenbosch University (ref: N13/04/056) approved the study.

### Analysis

Descriptive statistics were used to describe all variables. Participants were analysed according to the credentialing status (credentialed versus non-credentialed group). Their perceived barriers to credentialing were also ranked. The most important barrier for each participant received a value of 1, the second most important barrier a value of 2 and so forth until the least important barrier received a value of 7. A mean ranking score was calculated for every barrier (denominator used was the number of participants that ranked that specific barrier); the top ranked barrier would therefore have the lowest mean score.

## Results

**Fig. 1:**
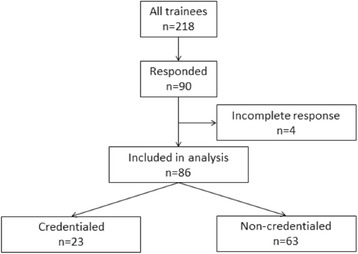
Flow diagram of study population

Two hundred and eighteen trainees were invited by e-mail to participate in the study. Ninety trainees completed the survey (response rate 41.3%); four surveys were excluded for being incomplete (Fig. 1). One fifth of trainees (*n* = 43, 19.7%) who entered the training programme prior to July 2013 credentialed successfully: 23 of them completed the survey. The medical specialties and base hospitals of respondents at the time of the survey are described in Table 1.

**Table 1:** Demographics and credentialing success rate of clinical ultrasound providers participating in the study

	All	Credentialed
	*n* (%)	*n* (%)
Medical speciality		
Emergency medicine	59 (68.6)	23 (39)
Internal medicine	10 (11.6)	0 (0)
Family medicine	5 (5.8)	0 (0)
Other (surgery, anaesthetics, critical care, general practitioners)	12 (14)	0 (0)
Place of work		
Central academic hospital	28 (32.6)	6 (21.4)
Regional hospital	25 (29.1)	8 (32)
District hospital	15 (17.4)	4 (26.7)
Other (primary health care, private practice, non-clinical management)	18 (20.9)	5 (27.8)

All 23 successfully credentialed trainees were working in the speciality of emergency medicine, yet the credentialing success amongst the emergency medicine cohort was only 39% (registrars *n* = 20, 87%; junior consultant with less than 5 years’ experience *n* = 2, 8.7%; senior house officer in emergency medicine *n* = 1, 4.3%).

The greatest barrier to credentialing amongst all trainees was severe time constraints, followed by access-related barriers (e.g. limited trainer access) (Table 2).

**Table 2:** Top three ranked barriers according to ranked mean scores

All		Credentialed		Non-credentialed	
Rank	Barrier	Rank	Barrier	Rank	Barrier
1	Time constraints	1	Time constraints	1	Time constraints
2	Limited access to credentialed trainer	2	Difficulty to gather positive scans	2	Limited access to credentialed trainer
3	Difficulty to save images	3	Limited access to credentialed trainer	3	Limited access to ultrasound machine

**Fig. 2:**
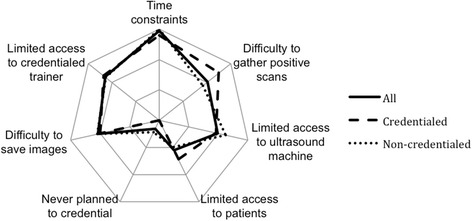
Perceived barriers to ultrasound credentialing (closest to centre is least important)

Access barriers (to trainers and ultrasound machines) were more dominant in the non-credentialed group whereas training logistics barriers (limited access to patients to log scans and difficulty obtaining scans with positive pathology) featured highly in the credentialed group (Fig. 2).

Alarmingly, 52.4% (*n* = 33) of the non-credentialed group performed on average more than three scans per week on patients where the scan result influenced their clinical management. However, 70% (*n* = 44) of the same group stated they were planning to complete the credentialing process in the near future.

## Discussion

The low credentialing success (19.7%) of the Cape Town clinical ultrasound training programme is concerning considering the time and resource investments made. The rate is significantly lower than six peer international training programmes, whose credentialing success ranged between 30.2 and 100%; however, all six studies were conducted in high resource settings [10–12, 14–16]. Of more concern, Cape Town’s credentialing success was most likely an overestimate of the national study population since the Cape Town sample represented 50% of the study population but also accounted for 90% of the national credentialing success.

The higher credentialing rate in the emergency medicine cohort could be explained by the 2009 CEMSA rule that only emergency medicine specialist training doctors (registrars) who successfully credentialed as clinical ultrasound providers are allowed to challenge the specialist training exit examinations. The ruling may also explain why only emergency medicine doctors completed the credentialing process (87% were registrars). However, the results also reflect poor uptake of ultrasound training (0%) amongst more experienced (greater than 5 years) emergency medicine consultants.

The most significant barrier to credentialing was severe time constraints. Trainees have limited spare capacity in their current work schedules and found the additional training time burden extremely challenging. This finding concurs with two studies that surveyed comparable target populations: Australian emergency medicine registrars and consultants reported ‘limited time availability’ (44.5%) as their highest ranked barrier, and American registrars and consultants also flagged ‘too many other demands on time’ as their greatest barrier amongst 71.3% of the trainees who failed to credential [10, 12]. The impact of limited access to resources on the time constraints barrier was not well described in any of the studies. To measure the positive effect on improving future time constraints when access barriers are alleviated will therefore need to be subjectively measured.

The highly ranked access barriers as perceived by the non-credentialing group concur with findings of a survey of health workers in 44 LMICs [9]. However, three studies conducted in much higher resourced settings that also trained over long distances and at multiple hospitals reported access to trainers and machines as their most important barriers after time constraints [7, 10, 14, 15]. All these training programmes had low credentialing success rates (30.2 to 44.9%) but still higher than Cape Town’s. Training programmes that divided their training capacity between only a few hospitals with a proper trainer and ultrasound machine access had the highest credentialing outcomes (67.7 to 100%) [12, 16]. Their trainees reported logistical barriers, related to their training programme curriculum, as their highest perceived barriers to credentialing.

Cape Town’s credentialed group experienced a combination of access (trainers and machines) and logistic barriers (difficulty to find patients with pathology to scan) as most important. All the credentialed study participants were emergency medicine doctors. The majority of them were based at relatively well-resourced hospitals for LMIC. They had better access to trainers and ultrasound machines than their peer trainees in other specialties. Stratifying the doctor’s hospital base to their credentialing success suggested that higher resourced hospitals with better access to ultrasound machines and trainers had better credentialing outcomes (refer to regional and district hospitals) (Table 1). Emergency medicine, a relatively new medical specialty in South Africa, is not yet well established at Cape Town’s central academic hospitals resulting in limited trainers and ultrasound machine availability for the trainees who were stationed there.

The finding that more than half of the study’s trainees continued to perform ultrasound scans on their patients despite not being credentialed as CUS providers is not unique. Two studies reported similar results from doctors in Australia and New Zealand [10, 17]. Doctors performing ultrasound on patients without completing their credentialing open themselves to significant liability risks irrespective of the frustration the perceived barriers may cause them [18]. In fact, such actions are deemed as fraudulent and could result in doctors being barred from further clinical practice [18].

The impacts of the study’s limitations were reduced in accordance with the selected study design. Regular reminders were sent to bolster the survey’s participant numbers to reduce the non-responder bias impact on the results. The survey’s eventual 41.3% response rate was higher than peer surveys (9.6–15%), and close to the 48% achieved by Shah et al., in studies that targeted similar ultrasound trainee study populations [9–11]. Trainees with a special interest in CUS were probably more likely to respond, which introduced responder bias, but stratifying the respondent surveys according to their credentialing success reduced its impact. Barriers reported by the non-credentialed group should be more reflective of the study population due to the high percentage (80.3%) that failed to credential. The low credentialing rate amongst the non-emergency medicine doctors could be explained by the dominance of emergency medicine representation within the training faculty and the fact that the CUS curriculum includes both trauma- and medicine-related module applications.

Training doctors to become competent in CUS is an expensive investment for any health system. The cost of purchasing and maintaining ultrasound machines for training has direct budget implications. Providing CUS trainers from an already scarce clinician pool has service delivery impacts. Credentialing success is a measurement of the investment return. It is essential to identify and analyse the barriers that reduce Cape Town’s credentialing success (19.7%) to less than that of its peer groups, so that targeted solutions can be found. Cape Town’s training programme is burdened with low resources, multiple training centres and relatively long distances between training hospitals (rural hospitals outside Cape Town), all conspiring to exacerbate poor access. Novel solutions must focus on improving future trainees’ access to ultrasound machines and trainer feedback without adding to the high monetary and service delivery sacrifices already made.

Others have recommended the use of distance learning web-based education platforms to overcome the unique burdens of training ultrasound within LMIC settings [9]. Web-based education platforms can be integrated successfully into a traditional well-structured apprenticeship model curriculums based on hands-on and simulation training [19]. The combination may result in improved skills proficiency when performing certain ultrasound-guided clinical procedures if the web-based component is introduced early enough in the training of junior residents (registrars or specialist training doctors) [19].

Future research must focus on adapting such web-based education platforms to improve overall access where trainers can provide feedback on scans submitted by off-site trainees. The development of such a novel web-based learning platform, focussing on improving credentialing success, will need to be measured against its efficacy in reducing the impact of these access barriers throughout its development.

## Conclusions

Access barriers to ultrasound machines and certified trainers are more prevalent in our low-resource setting. Training over large distances and at multiple training locations compounded the access barriers experienced. Novel solutions are necessary to overcome these access barriers to eventually improve credentialing success.

## Additional file

Additional file 1: Survey questionnaire. (DOCX 91 kb)

## Abbreviations

CEMSA: College of Emergency Medicine of South Africa
CUS: Clinical ultrasound
EMSSA: Emergency Medicine Society of South Africa
HIC: High-income country
IFEM: International Federation for Emergency Medicine
LMIC: Low- and middle-income countries
