# Supplementary material for: Poor return on investment: investigating the barriers that cause low credentialing yields in a resource-limited clinical ultrasound training programme
Source: Int J Emerg Med. 2018 Feb 21;11:11. doi: 10.1186/s12245-018-0168-9 (PMC5821624; doi:10.1186/s12245-018-0168-9)
Supplement: Supplementary file 1 — Survey questionnaire. (DOCX 91 kb) [file 12245_2018_168_MOESM1_ESM.docx]

# Additional File

## Survey Questionnaire

**Thank you for taking the time to complete this survey!**

**All your answers are anonymous and will be kept confidential**

**Directions: Please choose one answer per question unless directed otherwise.**

1. **What was your highest academic qualification at the time when you attended the ultrasound introductory course**:

- Primary Graduate degree ex. MB ChB etc.
- Post-graduate diploma ex. DA, Dip PEC etc.
- Post-graduate Masters degree ex. MMed, MPhil etc.
- Post-graduate specialist college qualification ex. FCP, FCS, FCEM etc.
- Doctorate degree ex PhD, MD etc.
- Other- please specify:____________________________________________

1. **What is your current highest academic qualification?**

- Primary Graduate degree ex. MBChB etc.
- Post-graduate diploma ex. DA, Dip PEC etc.
- Post-graduate Masters degree ex. MMed, MPhil etc.
- Post-graduate specialist college qualification ex. FCP, FCS, FCEM etc.
- Doctorate degree ex PhD, MD etc.­­­­­­­­­­­
- Other- please specify:____________________________________________

1. **What was your job title when you completed the introductory course?**

- Intern
- Community Medical officer
- Medical Officer
- Junior Registrar (Year 1-2)
- Senior Registrar (Year 3-4/5)
- Subspecialist senior registrar
- Junior consultant less than 5 years experience
- Senior consultant more than 5 years experience
- Subspecialty consultant
- Other- please specify:____________________________________________

1. **What is your current job title at the time of completing this survey?**

- Intern
- Community Medical officer
- Medical Officer
- Junior Registrar (Year 1-2)
- Senior Registrar (Year 3-4/5)
- Subspecialist senior registrar
- Junior consultant less than 5 years experience
- Senior consultant more than 5 years experience
- Subspecialty consultant
- Other- please specify:___________________________________________

1. **What was your clinical area of work at the time when you attended the course?**

- Emergency Medicine
- Internal Medicine
- Surgery
- Paediatrics
- Intensive care
- Anaesthetics
- Family Medicine
- General Practitioner
- Other- please specify:____________________________________________

1. **Which clinical area are you working in at present?**

- Emergency Medicine
- Internal Medicine
- Surgery
- Paediatrics
- Intensive care
- Anaesthetics
- Family Medicine
- General Practitioner
- Other- please specify:___________________________________________

1. **Years of clinical experience after completion of your MBChB (or equivalent) at the time of the ultrasound course:**

- 2 years or less post primary medical qualification
- 3-5 years post primary medical qualification
- 6-9 years post primary medical qualification
- More than 10 years post primary medical qualification

1. **Type of facility where you are currently working most of your clinical time:**

- Primary Health Care (Community Health Centre, Other Clinic)
- District hospital
- Regional hospital
- Tertiary
- Private practice
- Non-Clinical (Management, Research, Education, outside medicine.
- Other:_________________________________________________________

1. **Geographical area where you currently work most of the time:**

- Western Cape
- Eastern Cape
- Northern Cape
- Kwa-Zulu Natal
- Gauteng
- Mpumalanga
- Limpopo
- Free State
- North-West
- Other country:__________________________________________________

1. **Have you successfully completed the credentialing process and obtained certification**

- **Yes**
- **No**

1. **The following are options of perceived barriers that made it difficult for you to complete your credentialing. Please RANK them in the order of importance (1 to 8) that you feel affected you the most.**

- I never planned to complete the ultrasound credentialing process (log the required scans & final assessment) at the time I attended the ultrasound introductory course.
- Limited access to ultrasound machines to log my scans
- Limited access to appropriate patients to practice my scans & log them
- Difficulty to gather certain prerequisite positive scans due to the scarcity of their pathology (example: Positive Abdominal Aorta Aneurism scans)
- Time constraints due to other commitments
- Difficulty to save my scanned images for log purposes for delayed review by a credentialed trainer
- Limited access to a credentialed trainer to approve my log scans on site & provide real time feedback
- Any obstacles not on list (please add and rank them) ____________________________________

1. **How often do you use point of care ultrasound in your daily clinical practice to enhance your clinical decisions (applicable to real patient management decisions)?**

- I never perform any scans on my patients
- 2 or less scans per week
- 3-10 scans per week
- 11-20 scans per week
- More than 20 scans per week

1. **If you have not credentialed yet, are you planning to challenge the ultrasound exam in the future?**

- Yes
- No
- Not applicable

1. **Any comments/suggestions regarding the training or credentialing process? Please elaborate:**

______________________________________________________________________________________________________________________________________________________________________________________________________________________________________________________________________________________________________________________________________________________________________________________________________

**THANK YOU FOR PARTICIPATING IN THIS SURVEY!**
